# Train-the-Trainers in hand hygiene facilitate the implementation of the WHO hand hygiene multimodal improvement strategy in Japan: evidence for the role of local trainers, adaptation, and sustainability

**DOI:** 10.1186/s13756-023-01262-8

**Published:** 2023-06-09

**Authors:** Hiroki Saito, Koh Okamoto, Carolina Fankhauser, Ermira Tartari, Didier Pittet

**Affiliations:** 1 Department of Emergency and Critical Care Medicine, St. Marianna University Yokohama Seibu Hospital, 1197-1, Yasashi-Cho, Asahi-Ku, Yokohama, Kanagawa Japan; 2 Faculty of Medicine, Institute of Global Health, University of Geneva, Geneva, Switzerland; 3 Department of Infectious Diseases, The University of Tokyo Hospital, Tokyo, Japan; 4 Infection Control Programme, Faculty of Medicine, University of Geneva Hospitals, Geneva, Switzerland; 5 Faculty of Health Sciences, University of Malta, Msida, Malta

**Keywords:** Hand hygiene, Infection prevention and control, Train-the-Trainers, Training, Implementation science, Multimodal strategy, World Health Organization

## Abstract

**Background:**

“Train-the-Trainers in hand hygiene” (TTT) is a standardized training to train infection prevention and control (IPC) practitioners with the aim to promote hand hygiene in health care according to the World Health Organization (WHO) multimodal improvement strategy. Little is known in the literature about the sustained impact of hand hygiene and IPC trainings adapted locally. The aim of this study is to describe the impact of three TTT courses conducted annually in Japan on the adoption of the WHO multimodal improvement strategy by local IPC practitioners who became a “trainer” after their first TTT participation as a “trainee”.

**Methods:**

Three TTT courses were conducted annually from 2020 to 2022 in Japan. A team “TTT-Japan” composed of more than 20 IPC practitioners who completed their first TTT participation adapted the original TTT program to reflect the local healthcare context in Japan, and subsequently convened the 2nd and 3rd TTTs. Pre- and post-course evaluations and post-course satisfaction surveys of the course participants were conducted to assess improvement in knowledge on hand hygiene and perception towards the course, respectively. Attitude and practice surveys of the TTT-Japan trainers were conducted to assess their perception and experience in hand hygiene promotion. The Hand Hygiene Self-Assessment Framework (HHSAF), a validated tool created by WHO to monitor the capacity of hand hygiene promotion at facility level, was applied at TTT-Japan trainers’ facilities to compare results before and after trainers’ engagement. We applied inductive thematic analysis for qualitative analyses of open-ended survey questions of the trainers’ attitude and practice surveys, and the Wilcoxon Sign Rank test for quantitive comparisons of pre- and post-data for the surveys and HHSAF.

**Results:**

158 Japanese healthcare workers participated in three TTT courses, the majority of whom (131, 82.9%) were nurses. Twenty-seven local trainers were involved in 2nd and 3rd TTTs. The scores of pre- and post-course evaluations significantly improved after the course (*P* < 0.001) and the improvement was consistent across all three TTTs. Post-course satisfaction survey showed that over 90% of the participants reported that the course met their expectations and that what they learned in the courses would be useful for their practice. Trainers’ attitude and practice survey showed that more than three quarters (76.9%) of the trainers reported that their experience as a trainer had a positive impact on their practice at their own facilities. Qualitative analysis of the trainers’ attitude and practice survey revealed that trainers appreciated continuous learning as a trainer, and group effort to promote hand hygiene as the TTT-Japan team. The HHSAF institutional climate change element at the trainers’ facilities significantly improved after their engagement as a trainer (*P* = 0.012).

**Conclusions:**

TTTs were successfully adapted and implemented in Japan, leading to sustained hand hygiene promotion activities by local trainers over three years. Further research is warranted to assess the long-term impact on local hand hygiene promotion in different settings.

**Supplementary Information:**

The online version contains supplementary material available at 10.1186/s13756-023-01262-8.

## Introduction

Hand hygiene is of paramount importance in infection prevention and control (IPC) in health care [1, 2]. Implementation science to promote hand hygiene for decades has been reflected in current IPC standards such as the WHO guidelines on core components for IPC programmes [3, 4]. However, recent reports on IPC highlighted that IPC training needs to be addressed as an area for improvement even in high-income countries [5], and the lack of standardized IPC curricula was identified as a gap [6]. “Train-the-Trainers in hand hygiene” (TTT) is a standardized training originally developed by University of Geneva Hospitals (HUG), a WHO collaborating centre on IPC and antimicrobial resistance (AMR), and has been conducted in multiple sites across more than 10 countries since 2016 [7]. The original TTT provided by HUG is a 3-day course of hand hygiene training based on the WHO multimodal improvement strategy, consisting of lectures and hands-on sessions including video materials and role-plays [7, 8]. It aims to train the “trainers” who can contribute further to hand hygiene promotion at their own healthcare facilities and surrounding areas. The effectiveness of TTT on knowledge dissemination and networking for hand hygiene has been proven before [7]. However, sustained TTT activities requiring adaptation at country level have not been reported systemically, with particular focus on a team of local trainers who completed an original TTT and adapted the materials for local use thereafter. In addition, healthcare facilities, and particularly IPC practitioners, have been heavily affected by COVID-19 globally since the end of 2019. COVID-19 has over-stretched facility resources and capacities despite an increased demand for better IPC and hand hygiene [2]. In this changing environment, there is a higher need for efficient and effective IPC trainings, including hand hygiene, under the restrictions from infection control perspectives during the COVID-19 pandemic. Furthermore, little is known in the literature about the IPC trainings adapted locally and modified to make training materials and methods more acceptable by local trainees. Here, we describe the impact of three TTT courses conducted annually from 2020 to 2022 in Japan with local adaptation by Japanese TTT trainees who subsequently became TTT trainers. The impact was collectively measured by assessment of knowledge improvement and satisfaction of participants, and the trainers’ perception towards TTT and actions for further hand hygiene promotion.

## Methods

### Country background: Japan

Japan was the 9th country in the world where TTT was conducted. Before TTT, Japan participated in a WHO global survey on IPC programmes conducted in 2019. According to this first nationwide survey, 42% of participating healthcare facilities reported that they provided interactive training such as simulation and/or bedside training [9]. Therefore, the need for a standardized and hands-on training was considered high in Japan, and a plan to hold TTT in Japan was made between the HUG IPC team and a focal point in Japan who had been trained in hand hygiene at HU--G, and had already provided TTT as a trainer in other countries together with the HUG team.

### Course organization and setting

The first TTT in Japan was held in January 2020. The HUG team was invited as trainers, and delivered the course mainly with English-Japanese translation by several Japanese physicians trained in infectious disease and IPC, and fluent in English. In addition, the standard TTT training materials were all translated into Japanese in advance. Otherwise, the program remained the same as the original TTT. Seventy-five Japanese healthcare workers (HCWs) participated from 22 out of 47 prefectures.

The second TTT in Japan was held in December 2021. A focal point in Japan recruited TTT “trainers” from the first TTT participants through a group email. Seventeen Japanese HCWs including physicians, IPC nurses and a physical therapist who completed the first TTT in addition to two physicians who assisted in the translation of the first TTT, and an IPC nurse who led a Japanese association for IPC nurses volunteered to become TTT “trainers” and formulated the team “TTT-Japan”. This team restructured the original TTT course into a 2-day on-line program for Japanese HCWs. The decision to hold TTT as a completely on-line format of training delivery was made due to COVID-19 measures in Japan where a gathering was still restricted, particularly among HCWs. Thus, the original 3-day program was shortened to the 2-day program because of tight work schedule for IPC practitioners. On-line breakout rooms were utilized to enhance small group learning during hands-on sessions of the course such as video materials of clinical scenarios for the “My 5 Moments for Hand Hygiene”, which were facilitated by a few trainers assigned to each group of five to six participants (i.e. trainees). The entire course was delivered in Japanese except an on-line lecture provided by a senior trainer from HUG (DP) with English-Japanese translation. Thirty-five Japanese HCWs participated from 16 prefectures.

The third TTT in Japan was held in November 2022. Six additional IPC nurses who completed the 1st or 2nd TTT, in addition to two IPC nurses in advanced level, joined the TTT-Japan team as trainers (27 TTT “trainers” in total as of November 2022, excluding a focal point in Japan). The program remained as a 2-day program with slight modification based on feedback from the 2nd TTT participants (Additional file 1). All trainees gathered on site while the contents were provided as a hybrid format of on-site and on-line training, that was mainly delivered on site in Japanese, except an on-line lecture provided by a senior trainer from HUG (DP). Fifty HCWs participated from 13 prefectures.

The three TTTs were supported by multiple academic societies and an official health organization in Japan to enhance visibility, credibility and scalability: the 1st TTT was organized by the Japanese Society of Infection Prevention and Control (JSIPC), the largest IPC academic society in Japan, with endorsement of the Japanese Association for Infectious Diseases and the Japanese Society of Intensive Care Medicine (JSICM). The 2nd and 3rd TTTs were co-organized by JSIPC, JSICM and National Health Organization (NHO). Through the recruitment process of the three TTTs, multiple channels were utilized including websites and group emails of the above-mentioned Japanese organizations and the TTT-Japan team. The course fee was 10,000 Japanese Yen (about 70 US Dollars or 65 Euros). The trainers received financial incentives according to policies set by the academic societies and NHO.

### Course content and local adaptation

The detail of the original 3-day TTT program was described elsewhere [7], and the same course program was applied to the 1st TTT in Japan. For the 2nd and 3rd TTTs, the local adaptation including the 2-day course program and on-line format was made by the TTT-Japan team in consultation with the HUG team who originally developed TTT. The course program was shortened to 2 days mainly by omitting lectures of advanced level such as validation process of hand hygiene observers and scientific literature review on hand hygiene, and focusing more on a hands-on session with video materials of clinical scenarios for the “My 5 Moments for Hand Hygiene” instead of an original role-play simulation session where participants play a scenario-based role game of patients and HCWs in various inpatient settings (Additional file 1).

For the 2nd and 3rd TTTs, presentation slides of the essential lectures were all translated into Japanese with some of the original photos replaced by photos taken in Japanese healthcare settings. A Japanese glossary of keywords for TTT was created to ensure consistency throughout the course. Some keywords were kept short for ease of memorization: for example, the five elements of the WHO hand hygiene multimodal improvement strategy such as “System Change” and “Training and Education” were all translated into Japanese with four Chinese characters (*Kanji*) each. For the 3rd TTT, some original video materials for “My 5 moments for Hand Hygiene” created by WHO were re-taken by the TTT-Japan team to adapt to Japanese healthcare settings and to minimize inconsistent judgement due to unclear images among trainers and participants who observe hand hygiene indications and actions in the videos. Lectures and hands-on sessions were delivered and/or led by different trainers to ensure trainers’ learning and to enhance information sharing from different health facilities of trainers with different healthcare settings.

### Questionnaire surveys

For TTT participants, pre- and post-course evaluations were conducted in the form of on-line and paper questionnaires, the majority of which were conducted on-line. These evaluations were distributed immediately at the beginning and the end of each TTT, respectively, and the completion of each exam by participants was confirmed in a timely manner. The content of these evaluations was essentially identical, replicating the original TTT material with English-Japanese translation [7]. These evaluations consisted of 22 questions across three domains, and aimed to evaluate the participants’ knowledge about hand hygiene key principles, WHO methodology for hand hygiene observation, and recognition and identification of “My 5 Moments for Hand Hygiene” described in clinical scenarios. Correct answers were scored as 1, incorrect answers as 0. The maximum score was 22. Additional questions were part of the questionnaire survey at the end of TTT to assess overall course satisfaction (“post-course satisfaction survey”).

After the 3rd TTT, an on-line questionnaire survey consisting of close- and open-ended questions was conducted by a focal point in Japan among the trainers of 2nd and 3rd TTTs to assess their attitude and practice toward hand hygiene promotion through changing a role from “a TTT trainee” to “a TTT trainer” ("trainers’ attitude and practice survey”) (Additional file 2). In addition, the trainers who worked for a health facility were asked to fill out the Hand Hygiene Self-Assessment Framework (HHSAF), a validated tool created by HUG and WHO to assess the status of hand hygiene promotion activities at healthcare facility level [10–12]. HHSAF was conducted as an on-line questionnaire. As a comparator (the baseline HHSAF prior to their first TTT participation as a trainee), some of the trainers had already submitted HHSAF prior to their participation in the 1st or 2nd TTTs as part of TTT training while others were asked to submit HHSAF as of the time prior to their first participation, if available. HHSAF consists of 27 indicators across the five elements of the multimodal improvement strategy based on the WHO guidelines (System Change, Training and Education, Evaluation and Feedback, Reminders in the Workplace, and Institutional Safety Climate) [13], with each subtotal maximum score of 100 and a maximum total score of 500.

Consent was obtained as all questionnaire surveys were answered, and data obtained from those who declined the consent was excluded from this study.

### Statistical and qualitative analysis

Descriptive analysis reporting frequencies and proportions were used for close-ended survey questions of the post-course satisfaction survey. The Wilcoxon Sign Rank test was applied to compare the scores of the pre- and post-course evaluations completed by the TTT participants as well as the scores of HHSAF at trainers’ facilities prior to trainers’ first TTT participation and after becoming a trainer. All statistical analyses were performed using SPSS Version 27.0 (SPSS Inc., Chicago, USA) with statistical significance of *p* value < 0.05.

Text data of open-ended survey questions for the trainer’s attitude and practice survey among the TTT trainers were analyzed using inductive thematic analysis as a qualitative analytic method [14]. MAXQDA (VERBI GmbH) was used for the analysis.

## Results

### Participant demographics

In total, 160 Japanese HCWs participated in three TTT courses among whom 158 agreed to participated in the study (two were excluded due to lack of consent) (Table 1). Of those, 131 (82.9%) were nurses and 16 (10.1%) physicians. Other participants included pharmacists (N = 3), laboratory technicians (N = 2), physical therapists (N = 2), an emergency medical technician, and employees of healthcare industrial companies (N = 3). Approximately 70% of the participants were affiliated with community hospitals; a quarter (24.1%) with university hospitals. More than half of the participants were from Kanto region where Tokyo is included, and the others were well distributed across all seven regions of Japan.

**Table 1:** Demographics of participants of three “Train-the-Trainers in hand hygiene” courses conducted in Japan between 2020 and 2022

	1st TTT (N = 74)	2nd TTT (N = 35)	3rd TTT (N = 49)	Total (N = 158)
*Profession*				
Nurses	62 (83.8%)	26 (74.3%)	43 (87.8%)	131 (82.9%)
Physician	9 (12.2%)	5 (14.3%)	2 (4.1%)	16 (10.1%)
Others*	3 (4.1%)	4 (11.4%)	4 (8.2%)	11 (7.0%)
*Affiliation type*				
University hospital	22 (29.7%)	6 (17.1%)	10 (20.4%)	38 (24.1%)
Community hospital	44 (59.5%)	28 (80.0%)	36 (73.5%)	108 (68.4%)
Others	8 (10.8%)	1 (2.9%)	3 (6.1%)	12 (7.6%)
*Affiliation location*				
Hokkaido/Tohoku	4 (5.4%)	1 (2.9%)	1 (2.0%)	6 (3.8%)
Kanto	48 (64.9%)	19 (54.3%)	14 (28.6%)	81 (51.3%)
Chubu	4 (5.4%)	4 (11.4%)	16 (32.7%)	24 (15.2%)
Kinki	6 (8.1%)	6 (17.1%)	17 (34.7%)	29 (18.4%)
Chugoku	4 (5.4%)	1 (2.9%)	1 (2.0%)	6 (3.8%)
Kyushu and Okinawa	8 (10.7%)	4 (11.4%)	0 (0%)	12 (7.6%)

*Others included pharmacists (N = 3), laboratory technicians (N = 2), physical therapists (N = 2), an emergency medical technician, and employees of healthcare industrial companies (N = 3)

### Pre- and post-course evaluations

**Fig. 1:**
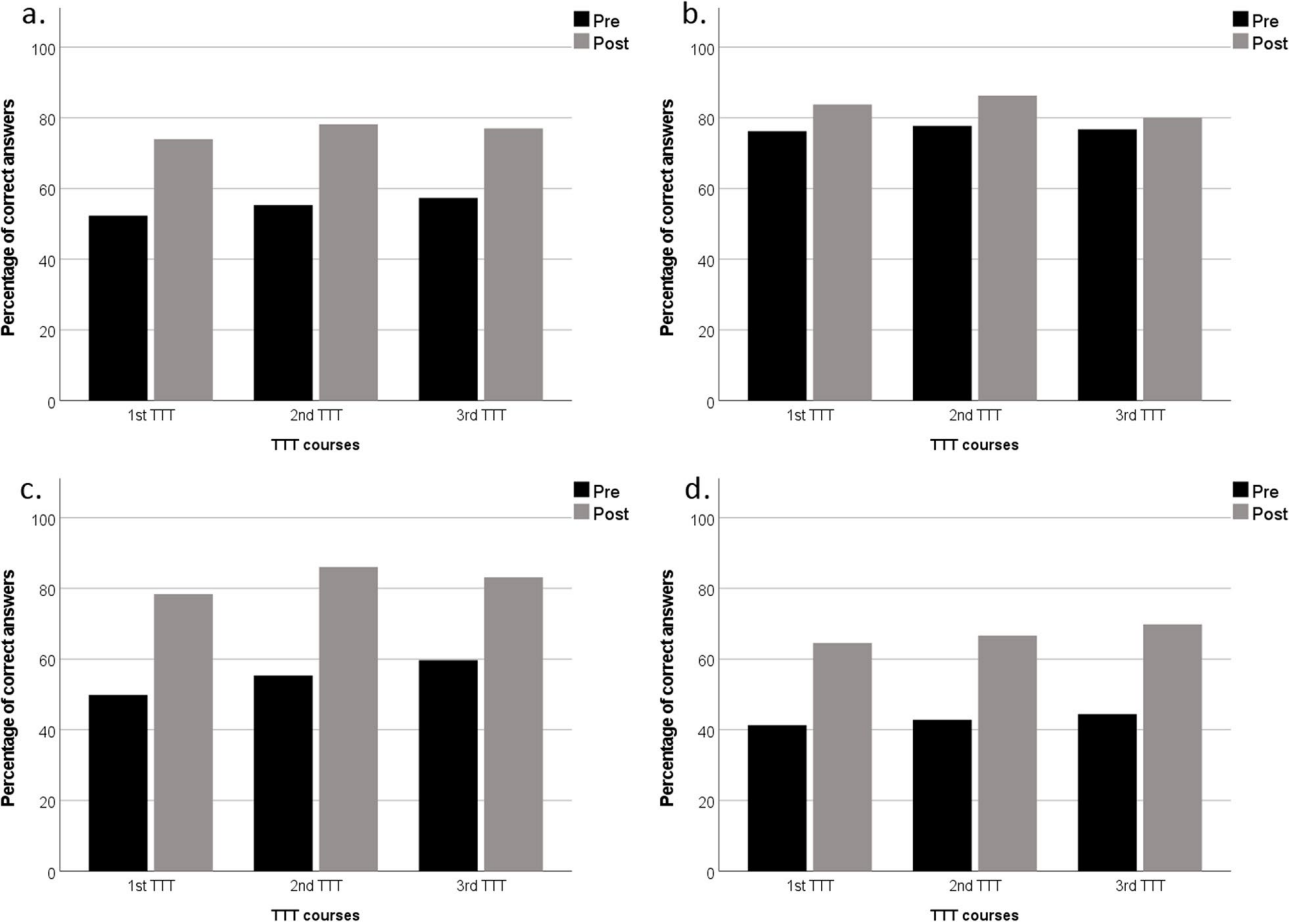
Results of pre- and post-course evaluations of Train-the-Trainers in hand hygiene. **a** Total scores. **b** Scores on Knowledge domain. **c** Scores on WHO Methodology domain. **d** Scores on Clinical Scenario domain. TTT: Train-the-Trainers in hand hygiene

Table 2 shows the results of the pre- and post-course evaluations among 158 participants. The total scores improved significantly after the course (*P* < 0.001). A statistically significant improvement was seen across all three domains. When looking at questions individually, the proportion of correct answers decreased in a clinical scenario describing the association between hand hygiene and glove use. The proportion of correct answers in a clinical scenario describing multiple simultaneous indications for “My 5 Moments for Hand Hygiene” after peripheral venous catheter removal remained low in both evaluations despite the significant improvement. There were no major differences of the evaluations between physicians and other participants, except that physicians scored higher in Question 20 (peripheral venous catheter) of post-course evaluation in 2020 (*P* = 0.004), and Question 22 (after touching patient surroundings) of pre-course evaluation in 2022 (*P* = 0.014). Overall, the statistically significant improvement was consistent across the three TTTs (Fig. 1).

**Table 2:** The results of the pre- and post-course evaluations among 158 participants in three “Train-the-Trainers in hand hygiene” courses

Questions	Correct answers (%) (pre-course)	Correct answers (%) (post-course)	*P* value
Total	54.5	75.8	< 0.001
Knowledge (5 questions)	76.7	83.2	< 0.001
Healthcare-associated infections	75.3	75.3	1.000
Microbial transmission	55.7	72.8	< 0.001
Hand rubbing/hand washing	85.4	93.0	0.023
Alcohol-based hand rub	79.7	88.6	0.023
Glove use	87.3	86.1	0.705
WHO methodology (8 questions)	54.1	81.6	< 0.001
Multimodal improvement strategy	63.3	94.3	< 0.001
Hand hygiene indications	44.9	86.1	< 0.001
Hand hygiene opportunities	67.1	70.9	0.396
The patient zone	62.7	88.0	< 0.001
Hand hygiene actions	65.2	70.3	0.276
The simultaneous observation	23.4	57.6	< 0.001
Duration of observation session	27.2	88.0	< 0.001
Five moments	79.1	97.5	< 0.001
Clinical scenarios (9 questions)	42.6	66.7	< 0.001
Before touching a patient	23.4	70.3	< 0.001
Before clean/aseptic procedures	56.3	77.8	< 0.001
Before/after touching a patient	46.2	89.9	< 0.001
After/Before touching a patient	62.7	96.8	< 0.001
After touching a patient	20.9	68.4	< 0.001
Glove use	46.8	32.3	0.004
Peripheral venous catheter	3.8	17.1	< 0.001
Urinary catheter	71.5	84.2	0.003
After touching patient surroundings	51.9	63.3	0.036

*Wilcoxon sign rank test

### Participants’ post-course satisfaction

A total of 152/158 participants (96.2%) completed the post-course satisfaction survey (Table 3). A large majority of them reported satisfaction with the courses. Over 90% of participants reported that what they learned in the courses would be useful for their practice and would lead to improvement in patient outcomes. Similarly, the participants reported that they would recommend the course to their colleagues. Compared to the other structures and contents of TTTs, the satisfaction rate was slightly lower regarding the balance between lectures and hands-on sessions, the length of the entire course, ease of participation and support for hands-on sessions. However, all the four areas significantly improved in the 2nd and 3rd TTTs. There were no major differences of the post-course satisfaction survey between physicians and other participants, except that physicians were more satisfized with TTT materials than other participants (“Were the course materials satisfactory?”) (*P* = 0.003).

**Table 3:** Post-course satisfaction survey of Train-the-Trainers in hand hygiene

Questions	1st TTT (N = 74)	2nd TTT (N = 31)	3rd TTT (N = 47)	Total (N = 152)	*P* value
Did the course meet your expectation?	68 (91.9%)	31 (100%)	47 (100%)	146 (96.1%)	0.037
Do you think what you learned in this course will be useful for your practice?	71 (95.9%)	30 (96.8%)	46 (97.9%)	147 (96.7%)	0.845
Do you think what you learned in this course will improve your patients’ outcome?	63 (85.1%)	31 (100%)	45 (95.7%)	139 (91.4%)	0.095
Do you recommend this course to your colleagues?	64 (85.6%)	30 (96.8%)	46 (97.8%)	140 (92.1%)	0.043
Were the topic covered in the course satisfactory?	66 (89.2%)	31 (100%)	47 (100%)	144 (94.7%)	0.012
Was the balance between lectures and hands-on sessions appropriate?	45 (60.8%)	28 (90.3%)	40 (85.1%)	113 (74.3%)	< 0.001
Was the length of the entire course appropriate?	29 (39.2%)	23 (74.2%)	38 (80.9%)	90 (59.2%)	< 0.001
Were hands-on sessions easy to participate in?	32 (43.2%)	24 (77.4%)	38 (80.9%)	94 (61.8%)	< 0.001
Was the support for hands-on sessions satisfactory?	49 (66.2%)	29 (93.5%)	43 (91.5%)	121 (79.6%)	0.002
Were the course materials satisfactory?	60 (81.1%)	31 (100%)	47 (100%)	138 (90.8%)	0.003

*Wilcoxon sign rank test

### Trainers’ attitude and practice

Of all 27 trainers who were actively involved in the TTT-Japan team as a trainer at the time of the survey, 26 (response rate, 96.3%) completed the trainers’ attitude and practice survey (Table 4). All trainers who completed the survey participated in the 1st or 2nd TTT as a trainee, except four (15.4%) who joined as a trainer without completing the full TTT course given their already acquired knowledge and skills. Thirteen (50.0%) trainers participated as a trainer twice. Overall, more than three quarters (76.9%) of the trainers reported that their experience as a trainer had a positive impact on their practice at their own facilities; none reported a negative impact. Twenty-two (84.6%) trainers reported that they would recommend participation in TTT as a trainee to their colleagues; 17 (65.4%) reported that they would recommend their colleagues to become a trainer, part of the TTT-Japan team, once their colleagues completed TTT as a trainee.

**Table 4:** Trainers’ characteristics, and attitude and practice as a trainer (N = 26)

	N (%)
*Demographics*	
Participation as a trainee	
1st TTT	17 (65.4%)
2nd TTT	5 (19.2%)
Never	4 (15.4%)
Participation as a trainer (multiple choice possible)	
2nd TTT	20 (76.9%)
3rd TTT	21 (80.8%)
*Attitude and practice*	
Agreed that the experience as a trainer made some positive impact on their practice at their facilities	20 (76.9%)
Agreed that the experience as a trainer made some negative impact on their practice at their facilities	0 (0%)
Recommend participation as a trainee to colleagues	22 (84.6%)
Recommend participation in TTT-Japan team as a trainer to colleagues once training completed	17 (65.4%)

*TTT* Train-the-Trainers in hand hygiene

Inductive thematic analysis revealed mainly two themes: learning as a trainer and working on hand hygiene promotion together as a team. As the first theme, trainers “constantly” learned about hand hygiene through “standardized” educational material from TTT participation both as a trainee and as a trainer, reinforcing their “confidence” as a trainer and helping them to promote further hand hygiene at their own health facilities, and to reach out surrounding health facilities. They learned from “other trainers” (i.e. each other) about “how to train” TTT participants, the staff at their healthcare facilities and their students at their educational institutes. As the second theme, the TTT-Japan team provided trainers an opportunity to get connected to other trainers across Japan, who shared “common interests” and “understanding” about hand hygiene, and to work on hand hygiene promotion as a team, which was a “motivation” for many trainers to continue their activities at their own and surrounding facilities. Through the experience of working on TTT as a team across Japan, trainers were also keen to recommend to their colleagues at their own facilities to participate in TTT so that they could compose a team of TTT participants within their own facilities for a greater impact on hand hygiene promotion. These themes were summarized by a trainer sharing “[Involvement in the TTT-Japan team] led me to better understand how to train hand hygiene and the WHO multimodal improvement strategy. (…) In addition, getting to know teammates across the country who work on hand hygiene promotion through TTT activities was a big plus for me.” On the other hand, trainers also expressed their challenges on a balance between their regular work at their facilities and additional work for the TTT-Japan team as a trainer.

Of the 27 trainers, 13 (48.1%) completed the HHSAF at their facilities before their first participation as a trainee and after involvement in the TTT-Japan team as a trainer. The total scores as well as the scores of each element in HHSAF are shown in Table 5. The total score increased without statistical significance between the two periods. The score for Institutional Climate Change element significantly improved from 40 to 60 points (*P* = 0.012).

**Table 5:** The scores of hand hygiene self-assessment framework at trainers’ own facilities completed before their participation in Train-the-Trainers in hand hygiene as a trainee and after their involvement in the Train-the-Trainers Japan team as a trainer (N = 13)

	Pre median (interquartile ranges)	Post median (interquartile ranges)	*P* values
Total	305.0 (217.5–355.0)	345.0 (270.0–372.5)	0.249
*Elements*			
System change	90.0 (65.0–92.5)	85.0 (65.0–92.5)	0.461
Training and education	60.0 (32.5–70.0)	75.0 (45.0–90.0)	0.181
Evaluation and feedback	65.0 (40.0–73.8)	65.0 (50.0–75.0)	0.366
Reminders in the workplace	65.0 (42.5–80.0)	60.0 (47.5–85.0)	0.504
Institutional safety climate	40.0 (30.0–55.0)	60.0 (37.5–80.0)	0.012

*Wilcoxon sign rank test

## Discussion

Three TTT courses were successfully implemented over three years in Japan and adopted locally with participants well represented from all regions across the country, even under the changing environment due to the COVID-19 pandemic, promoting hand hygiene in healthcare according to the WHO guideline. Local trainers played a significant role in the subsequent TTTs after they participated in the 1st TTT or 2nd TTT. Our results suggest that TTTs conducted by local trainers and in different formats of training delivery including on-line only program were well accepted by participants and consistently improved participants’ knowledge on hand hygiene, though the study suggests further training or clarification may be warranted particularly about multiple simultaneous indications for “My 5 Moments for Hand Hygiene” and the relationship between hand hygiene and glove use. Furthermore, a serial evaluation of HHSAF at the trainers’ own facilities improved after their TTT participation, highlighting the sustained impact of the trainers on local adoption of the WHO multimodal improvement strategy.

Hand hygiene is reported to be the most important single measure to reduce the transmission of microorganisms/pathogens and healthcare-associated infections [1]. It has been promoted at healthcare facility level around the world [11]. including the implementation of the WHO multimodal improvement strategy [15–17], but effective strategies for “Training and Education”, one of the five elements of the strategy, are yet to be systemically determined [18]. While the importance of implementation science is emphasized to make IPC related guidelines and guidance more actionable [19], TTT originally developed by HUG has been reported to be a potential training method to address this issue and successfully implement the WHO multimodal improvement strategy [7]. Tartari et al. previously reported some countries as a success study case rolling out subsequent training courses organized by the TTT participants; however, its impact was not systemically reported [7]. The current study demonstrates that local adaptation of the original TTT by local trainers could further promote hand hygiene, both through disseminating knowledge and skills to new TTT participants, and trainers’ sustained implementation of and commitment to the WHO multimodal improvement strategy at their own facilities. The median HHSAF score among 13 study facilities prior to trainers’ involvement in the TTT-Japan team was 305, which is comparable to 302, the median score among 47 health facilities in Japan that participated in the 2019 WHO HHSAF global survey [11]. Although there is no follow-up data available following the 2019 survey, this study showed that the median HHSAF score of trainers’ facilities increased to 345 over two to three years. Such HHSAF improvement did not reach statistical significance possibly due to the small number of facilities (N = 13); however, our findings strongly suggest trainers’ strong dedication to hand hygiene promotion at their own facilities. Particularly, the HHSAF improvement in the Institutional Climate Change element at the trainers’ facilities supports significant organizational efforts at institutional level, such as leadership involvement.

It is unique that TTTs in Japan were conducted at the beginning of and throughout the COVID-19 pandemic. Hand hygiene compliance in health care was impacted by the pandemic across countries [20–24]. The original TTT, designed as a 3-day program, was adapted to a 2-day program for the 2nd and 3rd TTT in Japan mainly due to more restricted work environment in Japan and the increased workload among IPC practitioners during the COVID-19 pandemic. In particular, the 2nd TTT was conducted purely as an on-line format, which was never reported in the literature. A systematic review on training and education of HCWs during viral epidemics reported e-learning as a mode of training delivery in 6/47 studies; findings were mainly derived from Ebola virus disease response [25]. Another systematic review and a meta-analysis of 14 studies with randomized controlled design identified other interventions, such as educational games, to improve HCWs adherence with IPC guidelines for respiratory infectious diseases; however the number of studies was small and most showed low to moderate certainty of evidence [26, 27].

Both the 2nd and 3rd TTTs were mainly provided by local Japanese trainers, and proved that the participants’ knowledge improved to a similar extent of the 1st TTT provided by the HUG team. The 1st TTT was provided through English-Japanese translation, accompanied by the translated educational material on hands, while the 2nd and 3rd TTTs were essentially provided in Japanese except for a single lecture given by the HUG team. Despite the shorter program and different format of training delivery for the 2nd and 3rd TTTs compared to the original TTT, overall participants’ satisfaction remained high and some areas even improved in the 2nd and 3rd TTTs according to the post-course satisfaction survey. These findings suggest that TTTs could be adapted locally in close collaboration with the HUG team that provided the original TTT, under the initiative taken by local trainers, and have an impact on sustained hand hygiene promotion locally, meeting the objectives of the original “Train-the -Trainers in Hand Hygiene” for subsequent local adoption. The original TTT not only trained local IPC practitioners on hand hygiene, but also acted as a “catalyst” helping them organize as a team of trainers, who subsequently contributed to the local adaptation and adoption of the WHO multimodal improvement strategy. The qualitative analysis of trainers’ attitude and practice survey supports local trainers’ sustained commitment to hand hygiene promotion through working with other trainers as a team of TTT-Japan.

Our study has some limitations. First, although TTTs were conducted annually for three years in Japan, the longer term impact remains to be determined, particularly given that these TTTs were heavily impacted by COVID-19, which may limit the generalizability of the study findings. Second, the trainers who composed a team “TTT-Japan” were considered to be IPC practitioners who had already been motivated and committed to hand hygiene promotion prior to their TTT participation, and the impact of the TTT participants who didn’t join the TTT-Japan team on subsequent hand hygiene promotion at their facilities was not compared. Third, the local adaptation including the change in the TTT program duration and contents, the languages used, the format of training delivery from the original on-site to the 2nd on-line and 3rd hybrid of on-site and on-line provision occurred simultaneously; therefore, factors that contributed to improved knowledge on hand hygiene and sustainability across multiple TTTs, as well as satisfaction among the participants are not separately measurable. Forth, the impact of TTTs on areas of IPC programs other than hand hygiene is not measured. This secondary impact of TTT beyond the scope of hand hygiene may warrant further research. Fifth, the language translation of TTT training materials from English to Japanese was not formerly validated. As a first step, a Japanese glossary of standardized terminologies for WHO multimodal improvement strategy may be required.

In conclusion, TTT is a standardized universal approach to guide hand hygiene education. It was successfully implemented in Japan and led to sustained hand hygiene promotion activities through locally adapted approaches provided by IPC practitioners who became TTT “trainers”. The group of local trainers played a significant role in the local adaptation and dissemination of TTTs across Japan, as well as local adoption of hand hygiene promotion at facility level. Further research is warranted to assess the long-term impact of TTTs on local hand hygiene promotion in different settings.

## Supplementary Information

**Additional file 1**: Train-the-Trainers in Hand Hygiene course programs.

**Additional file 2**: Trainers’ attitude and practice survey.

## Data Availability

The datasets for the study are available from the corresponding author upon request.

## Abbreviations

AMR: Antimicrobial resistance
HCWs: Healthcare workers
HHSAF: Hand hygiene self-assessment framework
HUG: University of Geneva Hospitals
IPC: Infection prevention and control
JSICM: Japanese Society of Intensive Care Medicine
JSIPC: Japanese Society of Infection Prevention and Control
TTT: Train-the-Trainers in hand hygiene
