# Supplementary material for: Train-the-Trainers in hand hygiene facilitate the implementation of the WHO hand hygiene multimodal improvement strategy in Japan: evidence for the role of local trainers, adaptation, and sustainability
Source: Antimicrob Resist Infect Control. 2023 Jun 9;12:56. doi: 10.1186/s13756-023-01262-8 (PMC10250848; doi:10.1186/s13756-023-01262-8)
Supplement: Supplementary file 1 — Additional file 1: Train-the-Trainers in Hand Hygiene course programs. [file 13756_2023_1262_MOESM1_ESM.pdf]

Train the Trainers in Hand Hygiene Program (2020)

11th January	
8:00	Registration
8:30	Opening remarks
8:45	Introduction of lectures
9:00	Pre-course exam
9:45	Coffee break
10:00	Opening remarks by a team from Geneva University Hospitals
10:15	Global Burden of Healthcare Associated Infections
10:45	The start of Hand Hygiene promotion in Geneva and going Global – the story behind the success
11:45	WHO Multimodal Hand Hygiene Improvement Strategy
13:00	Lunch
14:00	Hands-on: video reviewing for "My 5 moments" for hand hygiene
16:45	Coffee break
17:00	A film "CLEAN HANDS"
18:00	Wrap-up of the day
12th January	
8:30	Overview of infection prevention and control, and antimicrobial resistance in Japan
9:00	Hand hygiene initiative for hand hygiene promotion in Japan
9:15	From "Clean Care is Safer Care" to "SAVE LIVES: Clean Your Hands" campaign
10:00	Coffee break
10:15	WHO Hand Hygiene Self-Assessment Framework (HHSAF)
11:15	Hands-on: How to complete the HHSAF?
12:15	WHO Infection Prevention and Control Assessment Framework (IPCAF)
13:00	Lunch
14:00	Hands-on: Simulation based clinical scenarios with role-play performance in groups (explanation)
14:25	Hands-on: Simulation based clinical scenarios with role-play performance in groups (preparation)
14:50	Hands-on: Simulation based clinical scenarios with role-play performance in groups (1)
16:30	Coffee break
16:45	Hands-on: Simulation based clinical scenarios with role-play performance in groups (2)
18:25	Wrap-up of the day

13th January	
8:30	What's new in Hand Hygiene?
9:15	Innovations in Hand Hygiene promotion worldwide
10:00	Compliance monitoring and validation of the observers
10:30	Coffee break
10:45	Hand hygiene excellence award
11:15	Hands-on: Experiences and challenges in Hand Hygiene<br>Participants' presentations and discussion (1)
13:00	Lunch
14:00	Hands-on: Experiences and challenges in Hand Hygiene<br>Participants' presentations and discussion (2)
15:25	Post-course exam
16:25	Coffee break
16:45	Closing remarks
17:15	Remarks from lecturers
17:45	Certificate giving ceremony
18:15	Wrap-up

Train the Trainers in Hand Hygiene Program (2021)

4th December	
8:45	Registration
9:00	Opening remarks
9:20	Pre-course exam
10:00	Coffee break
10:10	Global Burden of Healthcare Associated Infections
10:55	Coffee break
11:05	The start of Hand Hygiene promotion in Geneva and going Global “Adapt to Adopt”
11:55	Lunch & a film “CLEAN HANDS”
13:00	WHO Multimodal Hand Hygiene Improvement Strategy / Hand Hygiene Excellence Award
13:45	Coffee break
13:55	WHO Hand Hygiene Self-Assessment Framework (HHSAF)
14:40	Coffee break
14:55	Hands-on: How to complete the HHSAF?
16:20	Wrap-up of the day
5th December	
9:05	“My 5 moments” for hand hygiene
10:00	Coffee break
10:15	Hands-on: video reviewing for “My 5 moments” for hand hygiene
11:30	Q&A / discussion on “My 5 moments” for hand hygiene
12:00	Lunch & a film “CLEAN HANDS”
13:00	Compliance monitoring and validation of the observers
13:30	What’s new in Hand Hygiene?
14:15	Coffee break
14:25	Post-course exam
15:05	Coffee break
15:15	Hands-on: Experiences and challenges in Hand Hygiene<br>Participants’ presentations and discussion
16:20	From “Clean Care is Safer Care” to “SAVE LIVES: Clean Your Hands” campaign
17:20	Closing remarks
17:30	Wrap-up

Train the Trainers in Hand Hygiene Program (2022)

12th November	
8:30	Registration
9:00	Opening remarks
9:30	Pre-course exam
10:10	Global Burden of Healthcare Associated Infections
10:55	Coffee break
11:00	WHO Infection Prevention and Control Assessment Framework (IPCAF)
11:50	Lunch & a film "CLEAN HANDS"
13:00	WHO Multimodal Hand Hygiene Improvement Strategy
13:45	WHO Hand Hygiene Self-Assessment Framework (HHSAF)
14:30	Coffee break
14:40	Hands-on: How to complete the HHSAF?
16:20	Coffee break
16:30	Q&A / discussion on HHSAF
17:00	Hand Hygiene Excellence Award
17:30	From "Clean Care is Safer Care" to "SAVE LIVES: Clean Your Hands" campaign
18:30	Wrap-up of the day
13th November	
9:00	"My 5 moments" for hand hygiene
9:50	Hands-on: video reviewing for "My 5 moments" for hand hygiene
11:45	Q&A / discussion on "My 5 moments" for hand hygiene
12:15	Lunch & a film "CLEAN HANDS"
13:10	Hands-on: Experiences and challenges in Hand Hygiene<br>Participants' presentations and discussion
15:10	Coffee break
15:20	Post-course exam
16:00	Q&A / discussion on the overall course program
16:30	Closing remarks
16:40	Wrap-up
