# Supplementary material for: Train-the-Trainers in hand hygiene facilitate the implementation of the WHO hand hygiene multimodal improvement strategy in Japan: evidence for the role of local trainers, adaptation, and sustainability
Source: Antimicrob Resist Infect Control. 2023 Jun 9;12:56. doi: 10.1186/s13756-023-01262-8 (PMC10250848; doi:10.1186/s13756-023-01262-8)
Supplement: Supplementary file 2 — Additional file 2: Trainers’ attitude and practice survey. [file 13756_2023_1262_MOESM2_ESM.pdf]

Trainers' attitude and practice survey (Japanese-English translation version)

1. When did you originally complete the TTT course?
   - 1st TTT (2020)
   - 2nd TTT (2021)
   - Never completed as a trainee

2. When were you involved in TTT as a trainer? (multiple choices possible)
   - 1st TTT (2020)
   - 2nd TTT (2021)
   - 3rd TTT (2022)

3. Working as a TTT Japan trainer makes a positive impact on your daily work. Please mark 1 to 6 according to your agreement on the statement (1 being strongly disagree and 6 being strongly agree)

   a. Please describe the positive impact. (If none, insert "none" in the box)

4. Working as a trainer of TTT makes a negative impact on your regular work. Please mark 1 to 6 according to your agreement on the statement (1 being strongly disagree and 6 being strongly agree)

   a. Please describe the negative impact. (If none, insert "none" in the box)

5. Would you recommend your colleagues to participate in TTT as a trainer? Please mark 1 to 6 (1 being strongly NOT recommend and 6 being strongly recommend)

   a. Please describe the above reasons.

6. Would you recommend your colleagues to become a TTT Japan trainer? Please mark 1 to 6 (1 being strongly NOT recommend and 6 being strongly recommend)

    a. Please describe the above reasons.

7. Please describe how your experience as a trainer is utilized in your daily work and activities on hand hygiene and infection prevention and control at your own facility. (If none, insert "none" in the box)

8. Please describe how your experience as a trainer is utilized in your work and activities on hand hygiene and infection prevention and control at the surrounding facilities and area. (If none, insert "none" in the box)

9. Please describe how your experience as a trainer is utilized in your work and activities on hand hygiene and infection prevention and control other than your own facility, and the surrounding facilities and area. (If none, insert "none" in the box)

10. Please make any other comments if any
